# Personal Experience in the Study of Disease

**Published:** 1909-03-13

**Authors:** 


### Personal Experience in the Study of Disease.

It is a very passable clinical aphorism that a single entire case often furnishes the key to many fragments of cases. This, doubtless, is part of the secret of the superiority, for purposes of investigation, of hospital over private practice; since in the former case there is opportunity for more continuous observation of the patient. Indeed, instances are not wanting where rather important clinical discoveries have been made first by observant and experienced nurses, persons whose attendance on the sick is much more uninterrupted than that of any medical man. Even their privileges, however, are plainly inferior to those of a physician who himself suffers from some chronic but not altogether disabling illness, and whose natural disposition to a philosophic, objective outlook on circumstances inclines him to deliberate study of his own case. From earliest times, of course, there must have been instances of this, although many of them are lost and others obscured by the uncertainty which attends the identification of modern diseases from descriptions written in remote periods. Seneca's account of his own sufferings from angina pectoris is classical. He was not a physician, by the way, although, like one or two of the fathers of medicine, something of a physicist. For the best illustrations of the subject under notice one must consult the more recent literature of migraine. This disease is known to affect especially intellectual people, and furthermore the predominance of its symptoms over its physical signs makes the anamnesis peculiarly valuable. In point of fact, a host of medical self-observers have left records. Abernethy remarked humorously (as humour went then) that during hemianopia from this cause he could see no more of his own name than the "knee" (*ne*) and the "thigh" (*thy*). Other names are those of Fothergill; Parry, of Bath, perhaps the first discoverer of exophthalmic goitre; Airy, who introduced the term teichopsia, and was the son of Sir George Airy, the astronomer, likewise a sufferer from, and writer on, migraine; the French pathologist Lebert; and Wollaston. Du Bois Reymond based upon his own symptoms a study of the pathology of the cervical portion of the sympathetic. From migraine to gout is not a long step, and Sydenham's personal experiences probably inspired not only the graphic description of its acute form, but also his testy remark on the diet appropriate to it, that "more importance is to be attached to the desires and feelings of the patient . . . than to doubtful and fallacious rules of medical art." Self introspection has had a place, too, in the study of asthma. Separated by an interval of a hundred years, the asthmatics Floyer and Bree advanced two of the principal theories of its causation now current. Trousseau's asthma used to wake him at three o'clock in the morning; he collected similar instances of periodical recurrence of this affection and others allied to it. When the malady studied happens to be very rare, like the myotonia which Thomsen suffered from, its possessor and observer occupies a position of peculiar authority, as also in such a case as that of Hack, who, having an empyema of his maxillary antrum, and being a rhinologist to boot, obtained a distinct advantage, in one sense, over fellow workers—an advantage which he turned to good account. But even in quite common complaints extreme familiarity has proved of service. The elder Mr. Weller's dictum as to the association of width with wisdom is borne out by Cheyne's aphorisms on obesity, although hardly by the cases of Bennet, Andrew Clark, Grancher, and the great Laënnec—all of them authorities on tuberculosis and themselves tuberculous. Self-knowledge in a bodily sense may indeed have been the starting point of research in many a case quite unsuspected. Had not he himself made no secret of the fact in his lectures, no one would have known that a certain distinguished living investigator used to suffer from a low degree of blood-coagulability, and also another abnormality which had an even greater share of influence on his work.

